# Modification of photosynthetic electron transport and amino acid levels by overexpression of a circadian-related histidine kinase *hik8* in *Synechocystis* sp. PCC 6803

**DOI:** 10.3389/fmicb.2015.01150

**Published:** 2015-10-20

**Authors:** Ayuko Kuwahara, Satomi Arisaka, Masahiro Takeya, Hiroko Iijima, Masami Yokota Hirai, Takashi Osanai

**Affiliations:** ^1^RIKEN Center for Sustainable Resource ScienceYokohama, Japan; ^2^Department of Agricultural Chemistry, School of Agriculture, Meiji UniversityKawasaki, Japan

**Keywords:** cyanobacteria, genetic engineering, histidine kinase, photosynthesis, *Synechocystis*

## Abstract

Cyanobacteria perform oxygenic photosynthesis, and the maintenance of photosynthetic electron transport chains is indispensable to their survival in various environmental conditions. Photosynthetic electron transport in cyanobacteria can be studied through genetic analysis because of the natural competence of cyanobacteria. We here show that a strain overexpressing *hik8*, a histidine kinase gene related to the circadian clock, exhibits an altered photosynthetic electron transport chain in the unicellular cyanobacterium *Synechocystis* sp. PCC 6803. Respiratory activity was down-regulated under nitrogen-replete conditions. Photosynthetic activity was slightly lower in the *hik8*-overexpressing strain than in the wild-type after nitrogen depletion, and the values of photosynthetic parameters were altered by *hik8* overexpression under nitrogen-replete and nitrogen-depleted conditions. Transcripts of genes encoding Photosystem I and II were increased by *hik8* overexpression under nitrogen-replete conditions. Nitrogen starvation triggers increase in amino acids but the magnitude of the increase in several amino acids was diminished by *hik8* overexpression. These genetic data indicate that Hik8 regulates the photosynthetic electron transport, which in turn alters primary metabolism during nitrogen starvation in this cyanobacterium.

## Introduction

The unicellular cyanobacterium *Synechocystis* sp. PCC 6803 (hereafter referred to as *Synechocystis* 6803) is one of the most widely studied cyanobacteria owing to its natural transformation ability and its genomic information (Ikeuchi and Tabata, 2001; Kanesaki et al., 2012). Knockout and overexpression of genes of interest in *Synechocystis* 6803 enable the researchers to investigating the molecular mechanisms of photosynthetic electron transport in this cyanobacterium (Ikeuchi and Tabata, 2001; Osanai et al., 2011).

Like other algae and plants, *Synechocystis* 6803 possesses Photosystem I and II (PSI and PSII). PSII is a multi-protein complex, localized in the thylakoid membrane, which functions as a light-driven water:plastoquinone oxidoreductase along with the Mn_4_Ca cluster (Nixon et al., 2010; Barber, 2014). The core of the PSII complex is composed of D1 and D2 proteins encoded by *psbA* and *psbD*, respectively (Mulo et al., 2009). Two chlorophyll-binding proteins CP43 and CP47, encoded by *psbC* and *psbB* respectively, are included in the reaction center of PSII (Barber, 2014). Three extrinsic proteins, PsbO, U, and V, are located on the lumenal side of cyanobacterial PSII and function as an oxygenic evolving complex (Barber, 2014). At least 20 proteins are included in PSII, and its dimeric crystal structure in thermophilic cyanobacteria has been resolved at a resolution of 1.9 Å (Umena et al., 2011). PSI is also a large membrane protein complex catalyzing light-driven electron transfer from the soluble electron carrier plastocyanin located on the lumenal side, to ferredoxin, located on the stromal side (Amunts and Nelson, 2009). The PSI complex is organized as a trimer containing 12 protein subunits (Jordan et al., 2001). The central part of the PSI core complex is formed by a heterodimer of the large transmembrane proteins PsaA and PsaB (Amunts and Nelson, 2009). The stromal loops of PsaA and PsaB are associated with the three stromal proteins PsaC, PsaD, and PsaE, which interact with ferredoxin (Amunts and Nelson, 2009). Plastocyanin is bound with PsaF at the lumenal part of PSI (Karapetyan et al., 2014). PsaI and PsaL are essential components of a trimer complex in cyanobacteria (Karapetyan et al., 2014).

The regulatory mechanisms of photosystems have been intensively studied in cyanobacteria. The D1 proteins are the main PSII subunits damaged during photoinhibition, and therefore, turnover of D1 proteins is an important photoprotective mechanism (Nixon et al., 2010). Regulation of D1 proteins at translational and post-translational levels is indispensable for the maintenance of PSII (Silva et al., 2003; Kojima et al., 2009). Down-regulation of the PSI/PSII ratio under high-light conditions is another way to acclimate to the fluctuation of light intensity (Murakami and Fujita, 1991). A response regulator, RpaB, binds the promoter regions of PSI genes and activates their gene expression under low light conditions (Seino et al., 2009). RpaB functions as a repressor of high light inducible genes in *Synechocystis* 6803 and *Synechococcus* sp. PCC 7942 (Kappell and van Waasbergen, 2007; Seki et al., 2007). Not only light conditions, but also nitrogen starvation can change the ratio of PSII and PSI activities in unicellular cyanobacteria (Görl et al., 1998). Overexpression of *sigE*, encoding an RNA polymerase sigma factor involved in sugar catabolism, modifies respiratory, and photosynthetic activities under both nitrogen-replete and nitrogen-depleted conditions (Osanai et al., 2013). Thus, light and nutrient conditions refine photosynthetic electron transport, which is altered through transcriptional cascades in *Synechocystis* 6803.

One of distinctive characteristic of cyanobacteria is their possession of circadian clocks. The central circadian oscillator consists of three proteins, KaiA, KaiB, and KaiC, and their phosphorylation cycle and transcription and translation feedback loops are essential for the generation of circadian rhythms (Ishiura et al., 1998; Nakajima et al., 2005). SasA is a histidine kinase associated with KaiC in *Synechococcus* sp. PCC 7942 (Iwasaki et al., 2000). Hik8 is an ortholog of SasA in *Synechocystis* 6803 and *hik8* knockout or overexpression alters the gene expression and metabolite levels related to primary carbon metabolism (Singh and Sherman, 2005; Osanai et al., 2015). The involvement of Hik8 in primary metabolism is thus genetically demonstrated, but its involvement in photosynthetic electron transport has not been demonstrated. The expression patterns of photosynthetic genes exhibit circadian oscillation during day/night cycle (Kucho et al., 2005), indicating that photosynthetic electron transport is under the control of circadian-related proteins.

Here we report significant changes in the expression of genes related to photosynthetic electron transport as a result of *hik8* overexpression. Analyses of respiratory and photosynthetic activities and amino acid levels, show a histidine kinase-mediated regulation of photosynthetic electron transport and primary metabolism in this cyanobacterium.

## Materials and methods

### Bacterial growth conditions

A glucose-tolerant (GT) strain of *Synechocystis* sp. PCC 6803, isolated by Williams (1988), and the *hik8*-overexpressing strain, designated as HOX80 (Osanai et al., 2015), were grown in modified BG-11 medium (Rippka, 1988) containing 5 mM NH_4_Cl (buffered with 20 mM HEPES-KOH, pH 7.8). The GT-I strain, among GT substrains, was used in this study (Kanesaki et al., 2012). Liquid cultures were bubbled with 1% (v/v) CO_2_ in air and incubated at 30°C under continuous white light (ca. 50–70 μmol photons m^−2^ s^−1^). For nitrogen starvation, cells grown in modified BG-11 were transferred into BG-11_0_ medium (BG-11 medium without NH_4_Cl) by filtration. Growth and cell densities were measured at OD_730_ with a Hitachi U-3310 spectrophotometer (Hitachi High-Tech., Tokyo, Japan).

### Measurement of respiratory and photosynthetic activities

Chlorophyll levels of cells grown under nitrogen-replete conditions were determined by a methanol extraction method (Grimme and Boardman, 1972; Iijima et al., 2015a). Cells containing 10 μg chlorophyll were resuspended in 1 mL BG-11_0_ liquid medium, supplemented with or without 5 mM NH_4_Cl, and incubated at 30°C within the chamber of an Oxytherm Clark-type oxygen electrode (Hansatech Instruments, King's Lynn, UK). Cells were incubated in dark conditions with monitoring of oxygen consumption for 10 min. The rate of oxygen consumption in the final 3 min of incubation was used to calculate respiration activity. Total oxygen evolution was measured after addition of 10 μL of 1 M NaHCO_3_ and exposure to white light of 1050 μmol photons m^−2^ s^−1^. The rate of oxygen evolution was calculated for the final 3 min of the 7-min measurement period.

### Absorption spectra with an end-on type spectrophotometer

Cells were cultivated in modified BG-11 medium for 1 day (started from OD_730_ = 0.2), collected by filtration, and then, re-suspended in BG-11_0_ medium. Absorption spectra were measured with an end-on type spectrophotometer MPS-2450 (Shimadzu, Kyoto, Japan). The data were normalized with OD_730_ = 1.0.

### Chlorophyll fluorescence

Chlorophyll fluorescence was measured with an AquaPen-C AP-C 100 fluorometer (Photon Systems Instruments, Drasov, Czech Republic). Chlorophyll levels of cells grown under nitrogen-replete and nitrogen-depleted conditions were determined and cells were diluted to 0.3 μg mL^−1^ chlorophyll *a* in 2 mL BG-11_0_ medium supplemented with or without 5 mM NH_4_Cl. Chlorophyll fluorescence was measured in accordance with the manufacturer's instructions (protocol NPQ1) after dark adaptation for 5 min. The intensity of actinic light and pulse-saturated light was 300 and 1500 μmol photons m^−2^ s^−1^, respectively. The wavelength of actinic light and pulse-saturated light was 450 nm. The *F*_*m*_ value was obtained after addition of 10 μM DCMU. The values of the photosynthetic parameters were calculated as described previously (Campbell et al., 1998; Sonoike et al., 2001), except that far-red light was not used in the present experiment. The values of qP, qN, NPQ, and ΦII were calculated as (*F*_m_′ − *F*_s_)/(*F*_m_′ − *F*_o_′), 1 − [(*F*_m_′ − *F*_o_′)/(*F*_m_ − *F*_o_)], (*F*_m_′ − *F*_s_)/*F*_m_′, and (*F*_m_ − *F*_m_′)/*F*_m_′, respectively.

### RNA isolation and quantitative real-time PCR

RNA isolation was performed as described previously (Osanai et al., 2014). The cDNAs were synthesized with the SuperScript III First-Strand Synthesis System (Life Technologies Japan, Tokyo, Japan) with 2 μg total RNA. Quantitative real-time PCR was performed with the StepOnePlus Real-Time PCR System (Life Technologies Japan) in accordance with the manufacturer's instructions, using the primers listed in Table S1. The expression level of *rnpB*, which encodes RNaseP subunit B, was used as an internal standard.

### Amino acid analysis by gas chromatography mass spectrometry (GC-MS)

Cells were cultivated in modified BG-11 medium for 1 day (starting from OD_730_ = 0.2), and equal amounts of cells (50 mL cell culture with OD_730_ = 1.0) were harvested by rapid filtration. Nitrogen-starved cells were similarly collected by filtration after 4 h of cultivation in BG-11_0_ medium. Amino acids were quantified by GC-MS as previously described (Osanai et al., 2014). All the results are listed in Table S2.

## Results

### Alteration of oxygen evolution and consumption by *Hik8* overexpression

**Figure 1:**
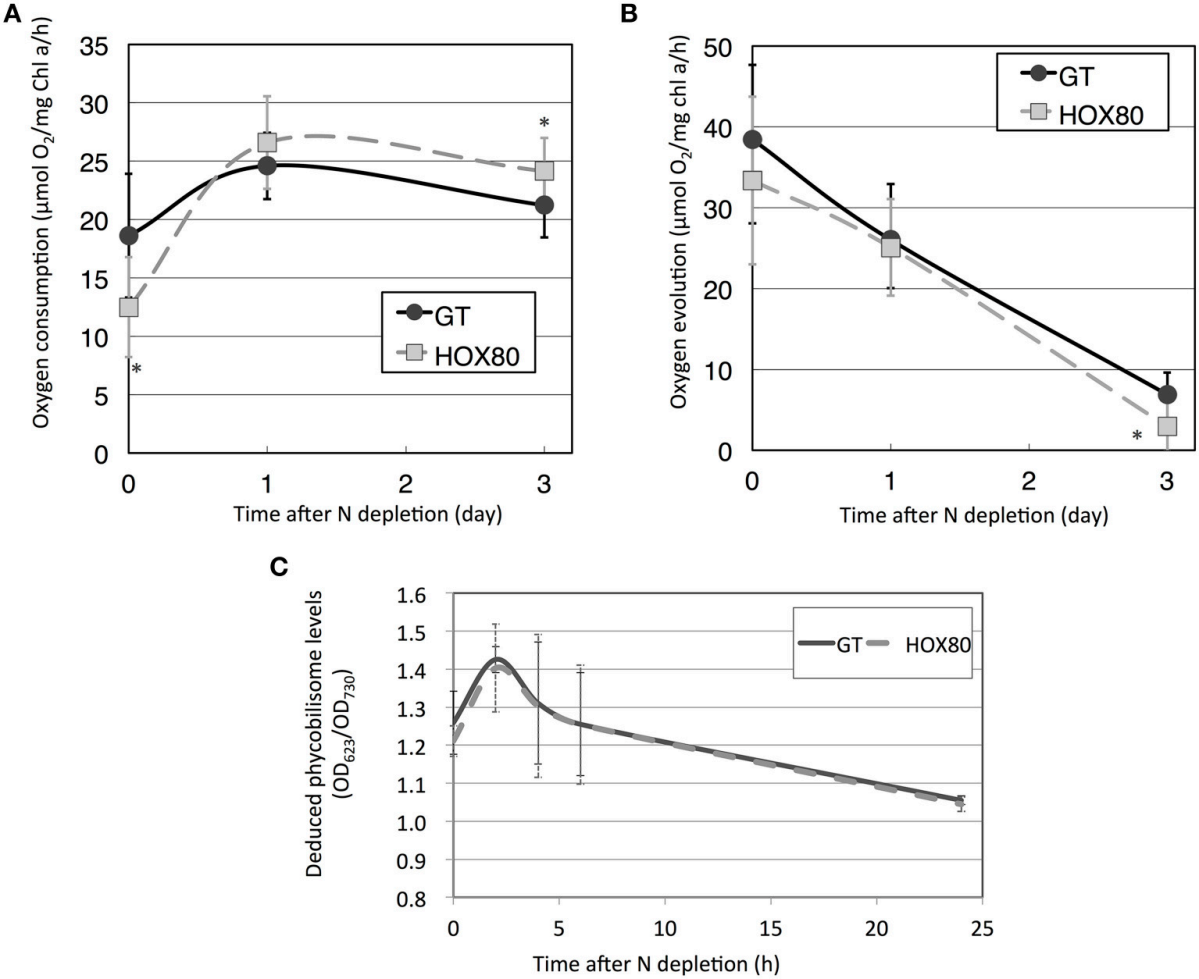
**Respiratory (A) and photosynthetic (B) activities of GT and *hik8*-overexpressing (HOX80) cells under nitrogen-replete and nitrogen-depleted conditions**. Data are the mean ± SD from seven independent experiments. Differences between GT and *hik8*-overexpressing cells were analyzed with Student's *t*-test. Asterisks denote statistical significance at ^*^P < 0.05. **(C)** The levels of OD_623_, representing absorption by phycobilisomes during nitrogen starvation. Data are the mean ± SD from eight independent experiments.

To study the role of Hik8 in photosynthetic electron transport, respiratory and photosynthetic activities were measured. Previous studies showed that Hik8 regulates the expression of genes related to sugar catabolism and the nitrogen-induced sigma factor *sigE* (Singh and Sherman, 2005; Osanai et al., 2015), and sugar catabolism in *Synechocystis* 6803 is particularly altered by nitrogen status (Osanai et al., 2006). Thus, we chose both nitrogen-replete and nitrogen-depleted experimental conditions. Respiratory activities of GT and HOX80 under nitrogen-replete conditions were 18.6 and 12.5 μmol O_2_ mg chl*a*^−1^ h^−1^, respectively, and thus, the respiratory activity of HOX80 was two-thirds of that in the GT strain (Figure 1A). The respiratory activity in GT increased by 1.3 times after 1 day of nitrogen depletion, whereas that in HOX80 increased by 2.1 times after 1 day of nitrogen depletion (Figures 1A,B). Photosynthetic activity was almost the same between GT and HOX80 under nitrogen-replete and nitrogen-depleted conditions for 1 day (Figure 1B). After 3 days of nitrogen depletion, respiratory, and photosynthetic activities in HOX80 were slightly higher and lower, respectively, than those in GT (Figures 1A,B). Changes in the color of the cultures during nitrogen starvation were similar between GT and HOX80. To confirm this, the absorption spectra were measured using an end-on type spectrophotometer. The transient increase and gradual decrease after prolonged nitrogen starvation in OD_623_ (which are the peaks representing phycobilisomes) were similar between GT and HOX80 (Figure 1C).

**Figure 2:**
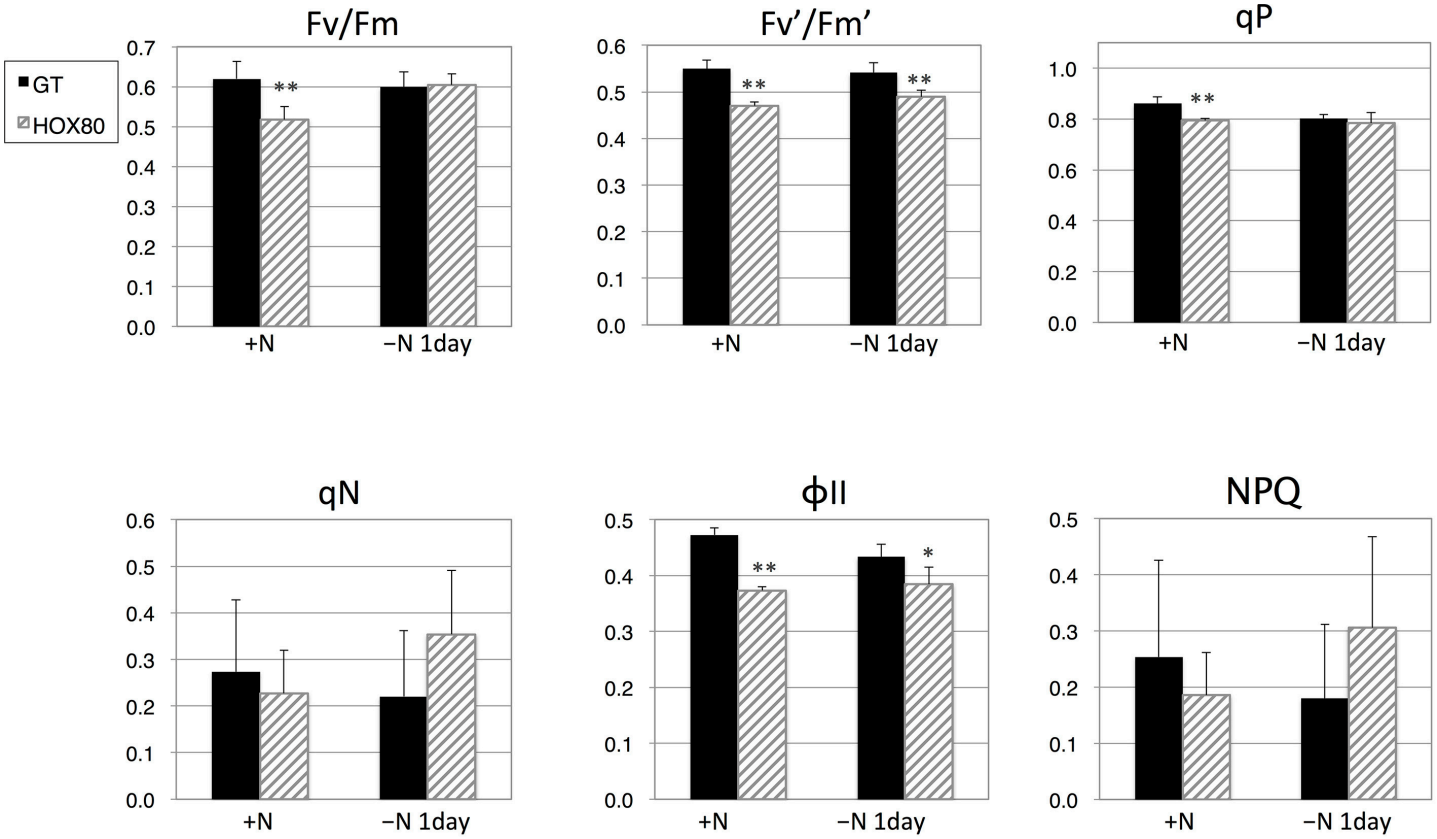
**Photosynthetic parameters for GT and *hik8*-overexpressing (HOX80) cells derived from chlorophyll fluorescence analysis**. Data are the mean ± SD from five independent experiments. Differences between GT and *hik8*-overexpressing cells were analyzed with Student's *t*-test. Asterisks denote statistical significance at ^*^P < 0.05 and ^**^P < 0.005.

Chlorophyll fluorescence was subsequently measured to determine the values of the photosynthetic parameters. The values of *F*v/*F*m (the maximal photochemical efficiency of PSII), *F*v′/*F*m′ (the photochemical efficiency of open PSII centers), qP (photochemical quenching), and ΦII (the effective quantum yield of electron transport through PSII) were decreased by *hik8* overexpression under nitrogen-replete conditions (Figure 2). The values of *F*v′/*F*m′ and ΦII in HOX80 were also lower than in GT under nitrogen-depleted conditions for 1 day (Figure 2).

### Alteration of the expression of genes related to electron transport

**Figure 3:**
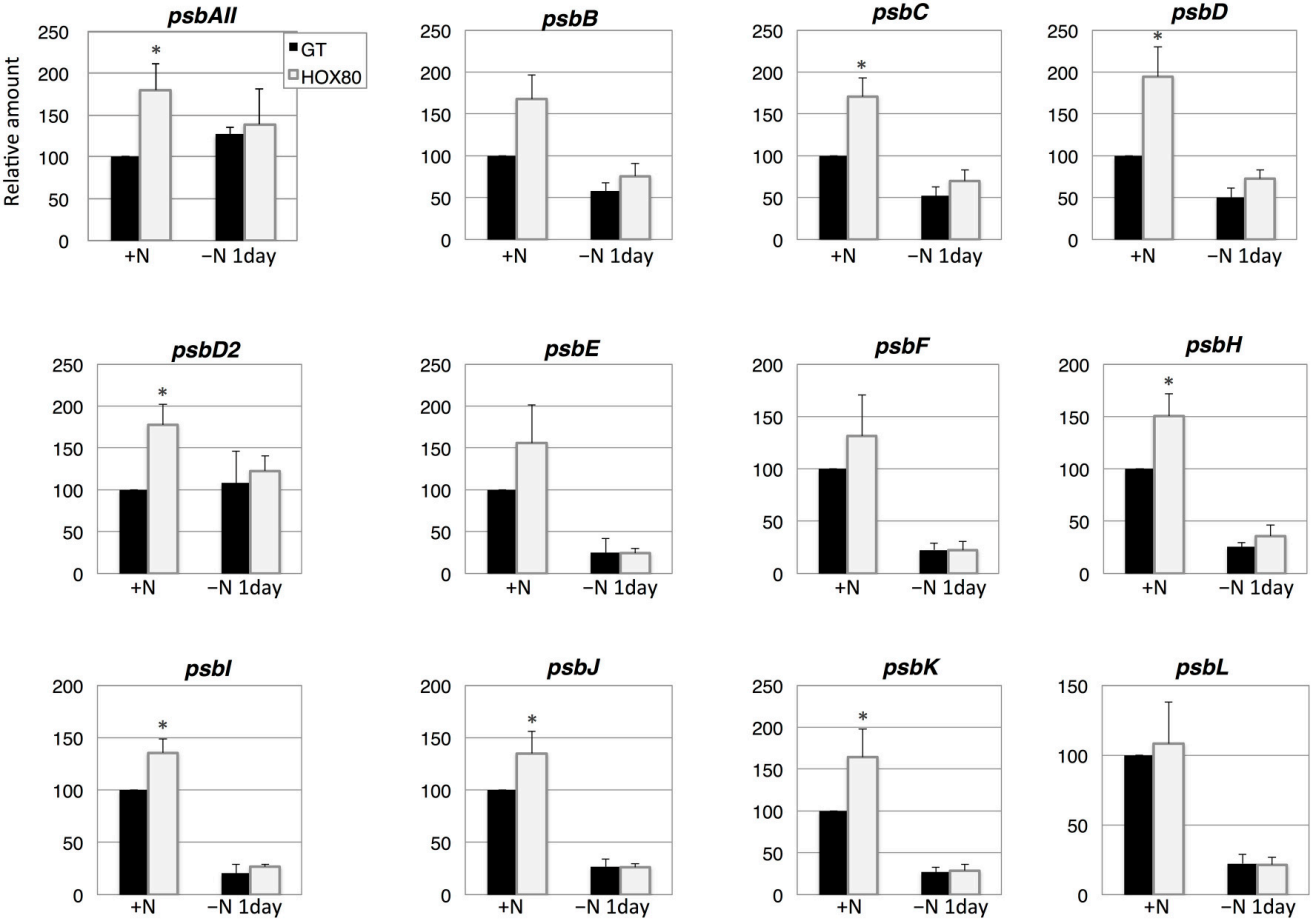
**Transcript levels of 12 genes encoding Photosystem II proteins in GT and *hik8*-overexpressing (HOX80) cells**. Data are the mean ± SD from independent experiments (*n* = 3~4). The levels were calibrated relative to the value obtained in the GT strain under nitrogen-replete conditions, which was set at 100%. Differences between GT and *hik8*-overexpressing cells were analyzed with Student's *t*-test. Asterisks denote statistical significance at ^*^P < 0.05.

**Figure 4:**
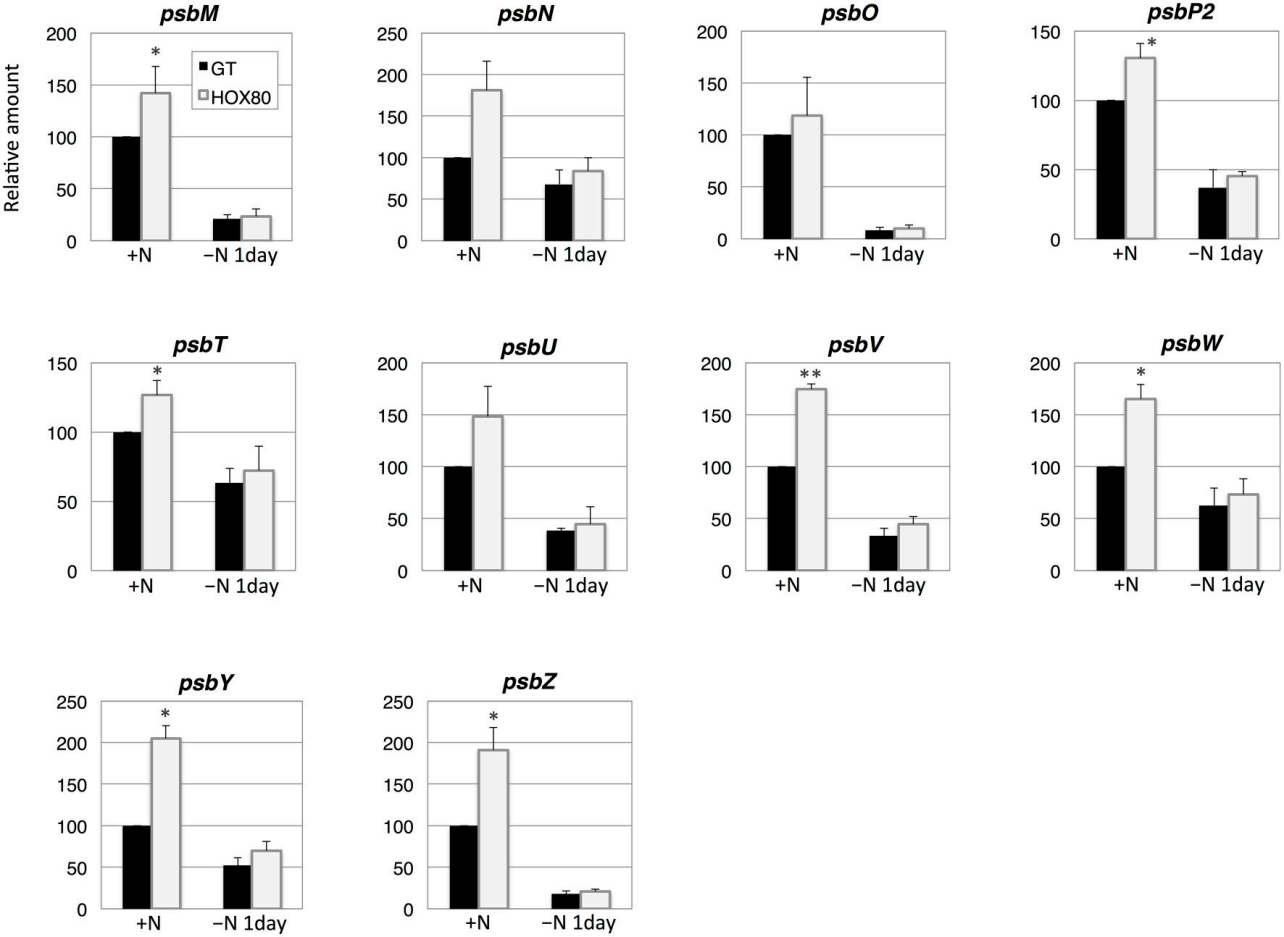
**Transcript levels of 10 genes encoding Photosystem II proteins in GT and *hik8*-overexpressing (HOX80) cells**. Data are the mean ± SD from independent experiments (*n* = 3~4). The levels were calibrated relative to the value obtained in the GT strain under nitrogen-replete conditions, which was set at 100%. Differences between GT and *hik8*-overexpressing cells were analyzed with Student's *t*-test. Asterisks denote statistical significance at ^*^P < 0.05 and ^**^P < 0.005.

Subsequently, the transcript levels of the genes related to electron transport were measured. The transcript levels of 15 out of 22 PSII genes increased as a result of *hik8* overexpression under nitrogen-replete conditions; this increase was statistically significant (Figures 3, 4). The transcript levels of genes encoding the reactive center of PSII (*psbAII, psbB, psbC, psbD*, and *psbD2*) increased more than 1.6 times by *hik8* overexpression under nitrogen-replete conditions (Figure 3). After nitrogen depletion for 1 day, the expression of all the genes except *psbAII* and *psbD2* was repressed in both GT and HOX80, and their levels were similar between the two strains (Figures 3, 4).

**Figure 5:**
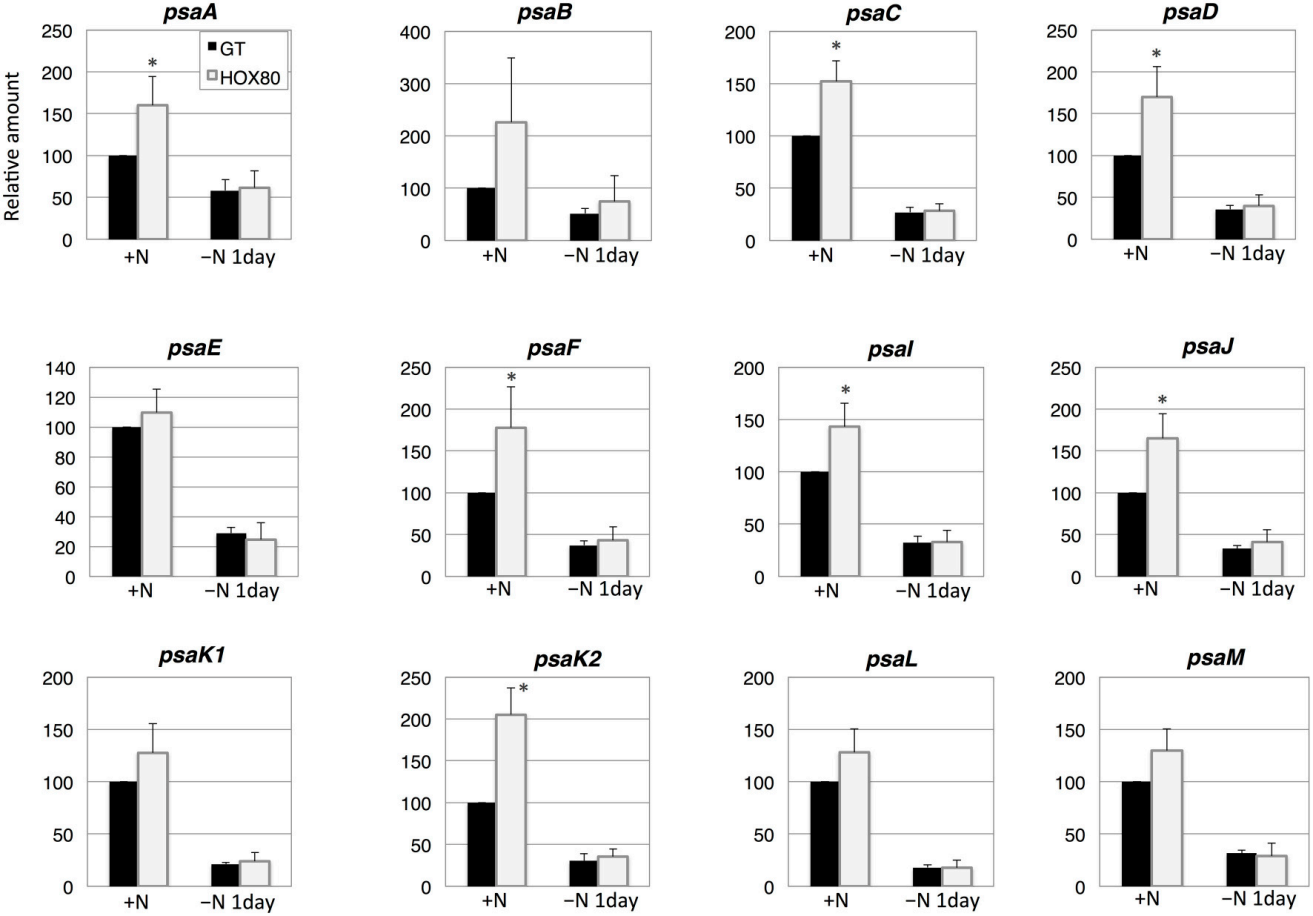
**Transcript levels of 12 genes encoding Photosystem I proteins in GT and *hik8*-overexpressing (HOX80) cells**. Data are the mean ± SD from independent experiments (*n* = 3~4). The levels were calibrated relative to the value obtained in the GT strain under nitrogen-replete conditions, which was set at 100%. Differences between GT and *hik8*-overexpressing cells were analyzed with Student's *t*-test. Asterisks denote statistical significance at ^*^P < 0.05.

The transcript levels of 7 out of 12 PSI genes increased as a result of *hik8* overexpression under nitrogen-replete conditions (Figure 5). The expression of all PSI genes was down-regulated during nitrogen starvation, and their transcript levels were similar between GT and HOX80 (Figure 5).

**Figure 6:**
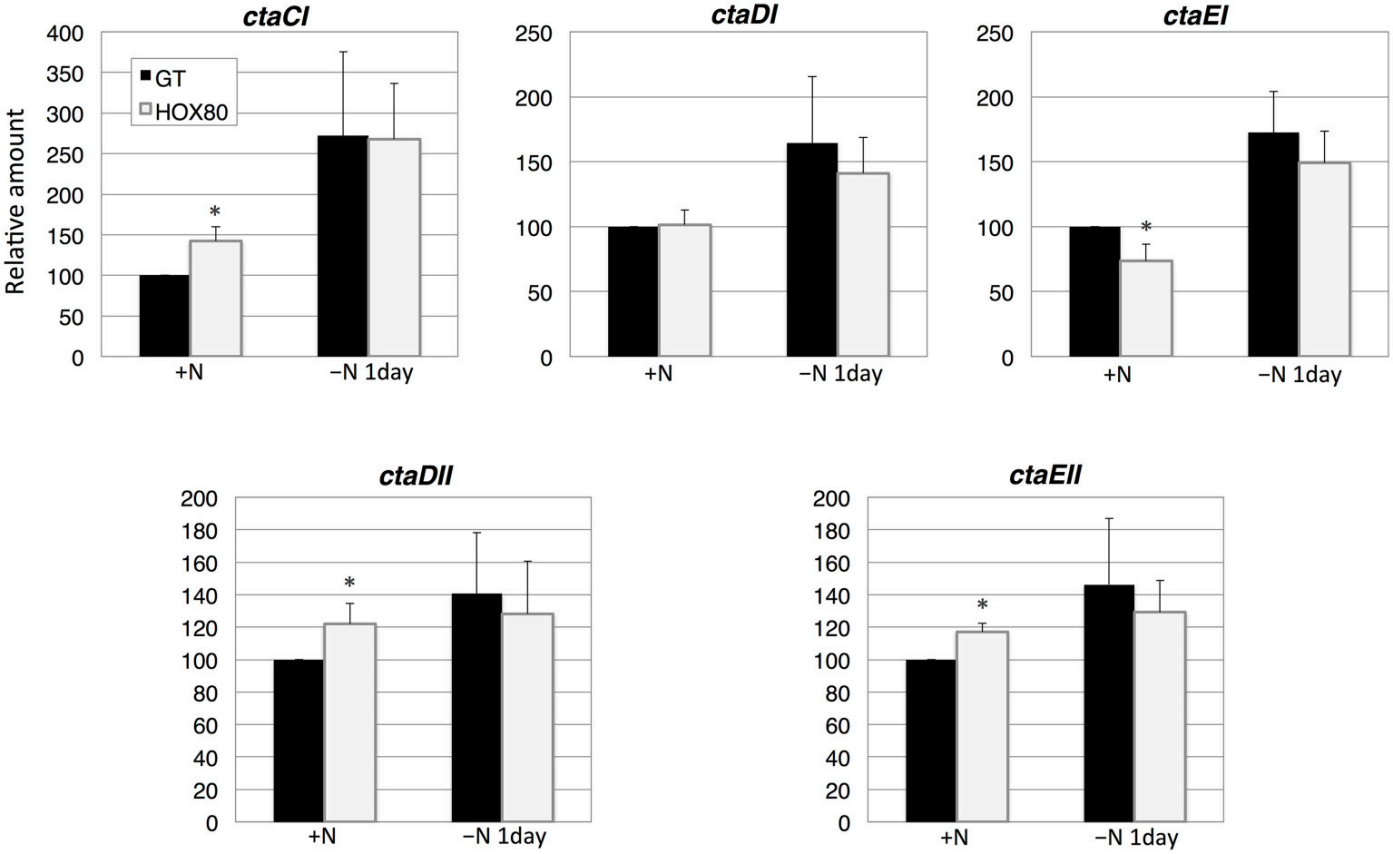
**Transcript levels of five genes encoding cytochrome *c* oxidase proteins in GT and *hik8*-overexpressing (HOX80) cells**. Data are the mean ± SD from independent experiments (*n* = 3~4). The levels were calibrated relative to the value obtained in the GT strain under nitrogen-replete conditions, which was set at 100%. Differences between GT and *hik8*-overexpressing cells were analyzed with Student's *t*-test. Asterisks denote statistical significance at ^*^P < 0.05.

The transcript analysis of five genes encoding the terminal cytochrome *c* oxidase showed that the transcript levels of *ctaCI, ctaDII*, and *ctaEII* increased by *hik8* overexpression under nitrogen-replete conditions, whereas the levels of *ctaEI* decreased (Figure 6). The expression of five cytochrome *c* oxidase genes was induced by nitrogen depletion, and the levels were similar between GT and HOX80 (Figure 6).

### Increase in amino acid levels after nitrogen depletion was abolished by *Hik8* overexpression

**Figure 7:**
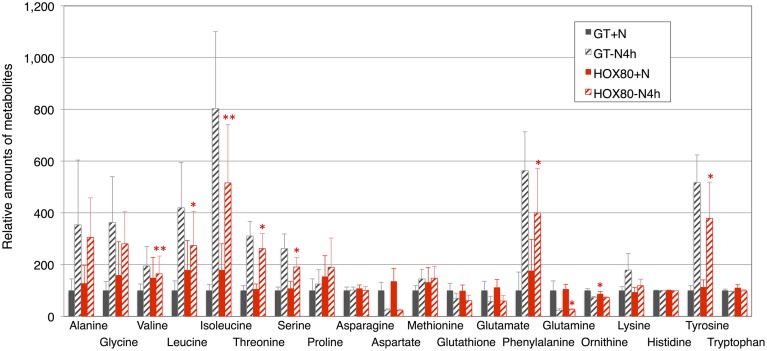
**Levels of 18 amino acids, ornithine, and glutathione**. Data represent means ± SD from four independent experiments. Levels were calibrated relative to that in GT grown under nitrogen-replete conditions (set at 100%). Asterisks indicate statistically significant differences between GT and HOX80 (Student's *t*-test; ^*^P < 0.05, ^**^P < 0.005).

Phycobilisome degradation during nitrogen-starved conditions is thought to provide amino acids as nitrogen sources (Richaud et al., 2001). The 18 amino acids, ornithine, and glutathione were quantified under nitrogen-replete and nitrogen-depleted conditions (Figure 7). The increases in valine, leucine, isoleucine, threonine, serine, phenylalanine, glutamine, and tyrosine by nitrogen depletion were abolished by *hik8* overexpression (Figure 7).

## Discussion

In this study, we examined the involvement of a histidine kinase Hik8 in photosynthetic electron transport, and our genetic analysis revealed that *hik8* overexpression modified the expression of PSII and PSI genes (Figures 3–5). Our analysis demonstrated that photosynthetic electron transport is regulated by a circadian-related protein in this cyanobacterium. Photosynthetic activity was partially decreased under nitrogen-depletion by *hik8* overexpression (Figure 1B), which is empirically consistent with a decrease in ΦII and *F*v′/*F*m′ in HOX80 (Figure 2). Previous study has showed that overexpression of *sigE* accelerates sugar catabolism and decreases the values of ΦII and *F*v′/*F*m′ (Osanai et al., 2013) and *hik8* overexpression activates sugar catabolism (Osanai et al., 2015). Thus, the degree of sugar catabolism and the values of ΦII and *F*v′/*F*m′ may be negatively correlated in this cyanobacterium. The value of *F*v/*F*m also decreased by *hik8* overexpression (Figure 2), indicating HOX80 strain contains aberrant PSII complexes. The expression of PSII and PSI genes was up-regulated as a consequence of *hik8* overexpression (Figures 3–5), and thus, the proper amount of photosynthetic transcripts and/or proteins was important for the activity of oxygen evolution. RpaA is a probable cognate response regulator of Hik8 and inactivation of *rpaA* resulted in a decrease in the monomeric PSI and D1 protein levels (Majeed et al., 2012). Although the direct involvement of Hik8 in photosynthetic electron transport is unclear, RNA-seq analysis has demonstrated that RpaA does not bind with the promoters of photosynthetic genes (Markson et al., 2013), nevertheless RpaA is important in adaptation to changes in light conditions (Iijima et al., 2015b). We here genetically showed that Hik8 manipulates the expression of genes related to PSI, PSII, and cytochrome *c* oxidases.

Several groups have shown a transient increase in amino acid levels after nitrogen depletion in *Synechocystis* 6803 (Hauf et al., 2013; Kiyota et al., 2014; Osanai et al., 2014). Amino acids containing additional nitrogen molecules (glutamine, glutamate, aspartate, and asparagine) decreased after 4 h of nitrogen depletion, whereas other amino acids increased (Osanai et al., 2014). Kiyota et al. (2014) divided amino acids into two groups: NblA-dependent and NblA-independent amino acids. NblA is a protein essential for the degradation of phycobilisome in cyanobacteria (Collier and Grossman, 1994). The NblA-dependent amino acids are glutamine, glutamate, glutathione, glycine, isoleucine, leucine, methionine, phenylalanine, proline, serine, threonine, tyrosine, and valine, and the NblA-independent amino acids are alanine, asparagine, lysine, and tryptophan (Kiyota et al., 2014). All eight amino acids whose induction was modestly decreased during nitrogen starvation by *hik8* overexpression (valine, leucine, isoleucine, threonine, serine, phenylalanine, glutamine, and tyrosine) are included in the NblA-dependent group (Figure 7). Since the phycobilisome degradation was similar between GT and HOX80 (Figure 1C), the reason for the difference of the amino acid levels between GT and HOX80 after nitrogen depletion was unclear. The interaction of photosynthetic electron transport and amino acid metabolism offers intriguing insights into the mechanisms of cell maintenance in cyanobacteria, and we suggest, from this study, that a circadian-related protein is important for this integrity. In this study, we found the metabolite levels and photosynthetic electron transport concomitantly altered in HOX80.

The regulation of photosynthetic genes by a histidine kinase named CSK has been demonstrated in *Arabidopsis thaliana* (Puthiyaveetil et al., 2010). CSK regulates the activity of bacteria-type RNA polymerase through control of the phosphorylation of sigma factor Sig1 (Puthiyaveetil et al., 2010). *Synechocystis* 6803 possesses multiple sigma factors, SigA–SigI (Osanai et al., 2008), and light-induced *psbAII*/*AIII* gene expression is reduced by *sigD* knockout (Imamura et al., 2003, 2004). Microarray experiments also indicate the involvement of SigD in the expression of photosynthetic genes (Summerfield and Sherman, 2007). Photosynthetic oxygen evolution is not affected by single or double knockout of group-2 sigma factors, but the double knockout of *sigB*/*sigD* leads to sensitivity to photoinhibition because of abolished up-regulation of *psbA* expression (Pollari et al., 2008, 2009, 2011). We previously showed that Hik8 positively regulates sigma factor SigE, and genetic modification of *sigE* alters photosynthetic electron transport (Osanai et al., 2013, 2015). In this way, regulation of photosynthesis by combinations of histidine kinases and sigma factors is conserved in both prokaryotic and eukaryotic photosynthetic organisms. Detailed analysis of the mechanisms controlling photosynthetic electron transport through transcriptional cascades is important, and it may also lead to an understanding of the regulatory mechanisms of primary metabolism in cyanobacteria.

### Conflict of interest statement

The authors declare that the research was conducted in the absence of any commercial or financial relationships that could be construed as a potential conflict of interest.
